# VES-13 and WHOQOL-bref cutoff points to detect quality of life in older adults in primary health care

**DOI:** 10.11606/S1518-8787.2019053000802

**Published:** 2019-03-27

**Authors:** Samira Monteiro Silva, Alfredo Nicodemos Cruz Santana, Nayhane Nayara Barbosa da Silva, Maria Rita Carvalho Garbi Novaes

**Affiliations:** I Secretaria de Saúde do Distrito Federal. Brasília, DF, Brasil; II Escola Superior de Ciências da Saúde. Curso de Medicina e Enfermagem. Hospital Regional da Asa Norte. Brasília, DF, Brasil

**Keywords:** Aged, Frail Elderly, Indicators of Quality of Life, Triage, classification, Sensitivity and Specificity, Primary Health Care

## Abstract

**OBJECTIVE:**

To determine Vulnerable Elders Survey (VES-13) and WHOQOL-bref cutoff points to detect poor quality of life (QoL) in older individuals.

**METHODS:**

This is a cross-sectional study, performed in all primary health care units in Samambaia, DF, Brazil. The data were collected from August 2016 to May 2017. The sample size of 466 older individuals treated in primary health care was obtained considering a 5% margin of error, 95% confidence level, 50% prevalence, and 20% possible losses, in a population of 13,259 older individuals. The subjects answered the VES-13 and WHOQOL-bref questionnaires. They were divided into 3 subgroups: poorQoL (older individuals with self-reported very poor or poor QoL AND very dissatisfied or dissatisfied with their health), goodQoL (very good or good QoL AND very satisfied or satisfied with Health) and indeterminateQoL (NOT belonging to poorQoL or goodQoL subgroups). A receiver-operating characteristic (ROC) curve was performed with poorQoL (case) *versus* goodQoL (control) to determine the cutoff score in VES-13 and WHOQOL-bref. A diagnostic test using these cutoffs was carried out in all older individuals (n = 466).

**RESULTS:**

The VES-13 and WHOQOL-bref cutoff points to detect poorQoL were ≥ 2 and < 60, respectively. The area under ROC curve of VES-13 and WHOQOL-bref was 0.741 (CI95% 0.659-0.823; p < 0.001) and 0.934 (CI95% 0.881-0.987; p < 0.001), respectively. In diagnostic tests, VES-13 showed 84% sensitivity and 98.2% negative predictive value, and WHOQOL-bref, 88% sensitivity and 99% negative predictive value.

**CONCLUSIONS:**

VES-13 score ≥ 2 and WHOQOL-bref score < 60 adequately detected poorQoL in patients treated in primary health care. Our data suggest that older individuals with these scores require special treatment such as geriatrics collaborative care to improve this scenario, considering QoL impact on mortality.

## INTRODUCTION

The evaluation of older people in primary health care (PHC) aims to prevent, to detect early and to treat diseases adequately. Consequently, these actions allow to maintain the best functionality and quality of life (QoL) and to reduce health vulnerability^1^ ^–^ ^3^, which is usually evaluated by a validated tool, called Vulnerable Elders Survey (VES-13), with scores ranging from 0 (best result) to 10 (worst result)^4^. In previous studies, individuals with a score ≥ 3 points in VES-13 showed worse outcomes^4^ ^–^ ^7^. Thus, the assessment of older adults treated in PHC using this tool is recommended, including by the Brazilian Ministry of Health^8^ ^,^ ^9^.

Considering that VES-13 is easily applicable and pertains to the routine care of older adults in PHC, it might be used in an interesting manner to evaluate QoL, and not only to predict functional decline and death^4^ ^–^ ^6^ ^,^ ^10^. However, to the best of our knowledge, there is no study validating the use of VES-13 to detect QoL in this specific population. In addition, the application of WHOQOL-bref (a well-known tool to predict QoL) is slower than VES-13, and is not included (according to the Brazilian Ministry of Health) as part of the routine care of older adults in PHC^9^ ^,^ ^10^. Finally, it is important to reinforce that QoL has a role in older adults mortality^11^ ^–^ ^13^.

Regarding QoL, it is usually evaluated using specific and validated tools, and one of the most often used is the WHOQOL-bref. This tool has a score that ranges from zero (the worst QoL) to 100 (the best)^10^ ^,^ ^14^ ^,^ ^15^. However, to our knowledge, only one study has proposed a cutoff point to detect QoL for older adults treated in PHC^15^.

Thus, the aim of this study was to estimate the cutoff point for VES-13 and WHOQOL-bref tools to detect QoL in older adults treated in PHC.

## METHODS

This study had a cross-sectional design. It was carried out at PHC units in the municipality of Samambaia (Distrito Federal, Brazil) from August 2016 to May 2017. For this study, the sample size was obtained by an estimate based on the total older population (in Samambaia) of 13,259, considering a margin of error of 5%, confidence level of 95%, and prevalence of 50%, thus obtaining a value of 388 individuals. Twenty percent was added to the sample to compensate for possible losses, reaching a final sample size of 466.

Each PHC unit participated in the study with a number of older adults that was proportional to the older population under the responsibility of PHC in relation to the total older population of the city of Samambaia. This estimate was based on a formula described in another study and was carried out as follows: 466 × (older population of the PHC unit / older population of the city)^15^.

The inclusion criteria of the study were: individuals aged 60 years or older who were spontaneously seeking health care at PHC units. Exclusion criteria were: severe cognitive impairment (defined as Mini-Mental State Examination score ≤ 9), refusal to participate in the study, or not signing the Informed Consent form. These criteria are in line with a previously published study^15^.

Previously trained researchers interviewed the patients who fit the criteria in the municipality of Samambaia. These interviews occurred in the morning or afternoon (according to the researcher's availability, as similarly described in other study on QoL by Silva et al.^15^, and were carried out in a standardized manner, using a data collection questionnaire. Such data were selected according to previous studies on older adults, such as the SABE study (an important cohort of older individuals in the city of São Paulo, Brazil)^16^ ^–^ ^18^.

Data on sociodemographic factors, comorbidities, geriatric syndromes, chronic medication use, health vulnerability and QoL were collected. Regarding sociodemographic variables, they were: age; gender; ethnicity; self-perception of the importance of religion in the patient's life; level of schooling; self-perception of sufficient income to meet their daily needs; marital status; and living alone or with someone^16^ ^–^ ^18^.

Data on comorbidities, geriatric syndromes and chronic medication use were: chronic pain; falls in the last 12 months; urinary incontinence; self-reported depression, diabetes mellitus, systemic arterial hypertension, chronic pulmonary disease (asthma or chronic obstructive pulmonary disease), heart disease, or cerebrovascular disease (stroke or transient ischemic attack) diagnosed by a physician; and chronic medication use; all in accordance with definitions used in previous studies^16^ ^–^ ^18^.

Health vulnerability and QoL were also assessed. For the first, the VES-13 tool (previously validated for use in Brazil) was used; and for QoL, the WHOQOL-bref tool was employed (also validated for use in Brazil)^8^ ^,^ ^10^. The VES-13 has 13 items (including age, physical activity limitations, limitations regarding basic and instrumental activities of daily life) addressing one domain^8^. Its score ranges from zero (best result) to 10 (worst result), with scores ≥ 3 points predicting worse outcomes (death and functional decline). Regarding WHOQOL-bref, it has 26 items addressing five domains, namely: overall, physical, psychological, social and environmental^10^. However, this study only used the overall domain, as in a previous study by Silva et al., to determine the WHOQOL-bref cutoff point for quality of life of older adults^15^.

Concerning VES-13, in order to determine the cutoff point for QoL prediction, a receiver-operating characteristic (ROC) curve was used. Two extreme subgroups were selected, based on the perception of QoL and health satisfaction^15^. Therefore, patients were divided into the poorQoL subgroup, comprising older adults who stated they had very poor or poor QoL and were very dissatisfied or dissatisfied regarding their health satisfaction^15^, and the goodQoL subgroup, comprising older individuals that said they had very good or good QoL and were very satisfied or satisfied regarding health satisfaction^15^. The other individuals who did not fit the two extreme subgroups described above were grouped as indeterminateQoL (and used in the diagnostic test)^15^.

After that, the VES-13 diagnostic test was applied to the poorQoL subgroup in relation to all the study patients (total n of 466; poorQoL PLUS goodQoL PLUS indeterminateQoL). Such calculation was performed in a similar way as previously described^15^. To facilitate understanding, a test for poorQoL is considered positive when the VES-13 score is ≥ the cutoff point derived from the ROC curve of the VES-13; and negative when the score is < the cutoff point.

Thus, sensitivity, specificity, positive predictive value and negative predictive value were estimated. Sensitivity is the percentage of older individuals in the poorQoL subgroup who were correctly detected by the positive test. Specificity is the percentage of individuals who did not belong to the poorQoL subgroup who were correctly detected by the negative test. Positive predictive value is the probability of having poorQoL when the test is positive. Finally, negative predictive value is the probability of not having poorQoL when the test is negative.

Regarding the WHOQOL-bref, to determine the cutoff point to detect QoL, the ROC curve was also used, as described for VES-13 (and as previously described)^15^. Two extreme subgroups were used, with one being poorQoL, and another, goodQoL^15^. After the cutoff point for WHOQOL-bref was defined in the ROC curve, the diagnostic test for the poorQoL group was performed in relation to all patients in the study (total n of 466, including poorQoL PLUS goodQoL PLUS indeterminateQoL)^15^.

A test was considered positive when the WHOQOL-bref score was < the cutoff point derived from the WHOQOL-bref ROC curve. A negative test was identified when the WHOQOL-bref score was ≥ the cutoff point. Therefore, its sensitivity, specificity, positive predictive value, and negative predictive value were estimated.

The statistical program Statistical Package for Social Science (SPSS) version 23.0 was used for the analyses described above. Additionally, the numerical variables are shown as means and standard deviations (SD); the categorical variables, as absolute numbers and percentages. Statistical significance was considered when p < 0.05.

This research was approved by the institutional Research Ethics Committee under number 1,667,060. The older individuals were informed about the purpose of this scientific research, and those who agreed to participate and met the eligibility criteria signed the Informed Consent form. Additionally, this study was carried out according to the Brazilian Human Research Ethical principles.

## RESULTS

The researchers assessed 475 older individuals for possible study enrollment. However, nine individuals were excluded (eight because they refused to participate in the study, and one because of a Mini-Mental State Examination score ≤ 9). Thus, the study included 466 older individuals, which agrees with the study design.

The sociodemographic and clinical characteristics of the sample (n = 466) are shown in Table 1. Some data are worth mentioning: 60.3% were women; 91.4% considered religion to be important; 60.5% had insufficient income; only 17.6% lived alone; 30% had suffered a fall, 30% reported urinary incontinence; 40.1% had diabetes; and 71.7% had systemic arterial hypertension.

**Table 1:** Sociodemographic and clinical characteristics of 466 patients treated in primary health care. Samambaia, DF, Brazil, 2017.

Sociodemographic and clinical characteristics		n
Age, years (SD)		68.2 (7.1)
Female (%)		281 (60.3%)
Ethnicity		
	Caucasian (%)	142 (30.5%)
	Mixed-race (%)	262 (56.2%)
	Black (%)	49 (10.5%)
	Native Brazilian (%)	2 (0.4%)
	Asian (%)	11 (2.4%)
Religion – important (%)		426 (91.4%)
Schooling level in years (SD)		4.6 (3.8)
Insufficient income (%)		282 (60.5%)
Marital Status – Married/Common-law marriage (%)		208 (44.6%)
Lives alone (%)		82 (17.6%)
Pain		304 (65.2%)
Depression		91 (19.5%)
Fall		140 (30%)
Urinary incontinence		140 (30%)
Diabetes		187 (40.1%)
Systemic arterial hypertension		334 (71.7%)
Chronic pulmonary disease		48 (10.3%)
Heart disease		102 (21.9%)
Cerebrovascular disease		55 (11.8%)
Number of medications (SD)		3.6 (0.1)

The results of VES-13 and WHOQOL-bref of the studied population (n = 466) are described below. The mean score was 2.3 (SD = 2.2) in the VES-13, and 61.2 (SD = 9.0) in the WHOQOL-bref overall domain, 62.9 (SD = 13.7) in the physical domain, 65.2 (SD = 10.9) in the psychological domain, 63.3 (SD = 13.3) in the social domain, and 53.3 (SD = 10.9) in the environmental domain.

Regarding the QoL subgroups, there were 25 older individuals in the poorQoL subgroup. There were 172 older individuals in the goodQoL subgroup, and 269 in the indeterminateQoL subgroup. Thus, the ROC curve of the VES-13 was performed and resulted in a cutoff point of ≤ 2 for the poorQoL subgroup in the studied individuals, with a good area under the curve (Table 2). The VES-13 diagnostic test (positive test for poorQoL when ≥ 2) showed very good sensitivity and excellent negative predictive value (Table 3).

**Table 2:** Results of the VES-13 and WHOQOL-bref ROC curves for older individuals with very poor (or poor) quality of life and very dissatisfied (or dissatisfied) with their health status. Samambaia, DF, Brazil, 2017.

Tools	Area under the ROC curve(95%CI)	Cutoff point	p
VES-13	0.741 (0.659–0.823)	≥ 2	< 0.001
WHOQOL-bref	0.934 (0.881–0.987)	< 60	< 0.001

VES-13: Vulnerable Elders Survey; ROC: receiver-operating characteristic; WHOOQOL-bref: World Health Organization Quality of Life - abbreviated version

**Table 3:** Results of the VES-13 (score ≥ 2) and WHOQOL-bref (score < 60) diagnostic tests for older individuals with very poor (or poor) quality of life and very dissatisfied (or dissatisfied) with their health status. Samambaia, DF, Brazil, 2017.

Tools	Sensitivity (%)	Specificity (%)	Positive predictive value (%)	Negative predictive value (%)
VES-13	84	49.4	8.6	98.2
WHOQOL-bref	88	66.4	12.9	99

VES-13: Vulnerable Elders Survey; WHOOQOL-bref: World Health Organization Quality of Life - abbreviated version

The results of the WHOQOL-bref ROC curve and the WHOQOL-bref diagnostic test for poorQoL were also significant. The ROC curve showed a value < 60 in the overall domain for poorQoL, with an excellent area under the curve (Table 2). Finally, the WHOQOL-bref diagnostic test (positive test for poorQoL when < 60 in the overall domain) showed both excellent sensitivity and negative predictive value (Table 3).

## DISCUSSION

The main finding of this study showed, for the first time, that the cutoff point ≥ 2 in the VES-13 had very good sensitivity and excellent negative predictive value for poorQoL. This result may bring important changes to clinical practice in PHC, since, until then, greater attention was given to older adults with a score ≥ 3 (greater risk of death and functional decline)^3^ ^–^ ^8^. However, since QoL is very important for older adults (including having a significant impact on the mortality increase), we suggest that older patients with a score ≥ 2 should be carefully assessed^19^ ^–^ ^21^.

This study also showed that the cutoff point < 60 in the overall domain of the WHOQOL-bref showed very good sensitivity, as well as an excellent negative predictive value for poorQoL. Until recently, only the study by Silva et al. had performed this analysis, suggesting a cutoff point < 60^15^. This result in our study was important to the external validation of this cutoff point in older individuals treated in PHC in a different city than the one previously studied by Silva et al.^15^

Regarding the characterization of the older population in our study, it was similar to that assessed in other researches. In relation to the study by Silva et al., the characteristics of age, sex, marital status and systemic arterial hypertension were especially similar; corroborating the fact that we found the same cutoff point in the WHOQOL-bref overall domain score for poorQoL^15^. Our sociodemographic and clinical data are also compatible with those of another population-based study, especially related to sex, marital status, and living alone^22^. Finally, the score of our study in the different domains of WHOQOL-bref was similar to the ones found by Tavares et al.^23^ Regarding all these aspects, there is a good possibility that future studies will corroborate our findings regarding the VES-13 and WHOQOL-bref cutoff points for poorQoL (external validation).

We emphasize, as study limitation, the use of a cross-sectional design. Consequently one can state that health vulnerability (measured by VES-13) is associated with QoL, but one cannot establish a causality correlation between them. However, this design was also used by Silva et al.^15^ Another limitation is that it was the first study to establish a cutoff point for VES-13 in poorQoL and the second to do it for WHOQOL-bref, which makes comparisons with other studies and populations very difficult.

We conclude that this study proposes as cutoff a value ≥ 2 in the VES-13 and a score < 60 in the overall domain of the WHOQOL-bref to detect older individuals with poorQoL treated in PHC. Thus, as a practical approach, older individuals with VES-13 score of two points or more would already require special treatment at the PHC unit, such as collaborative care or consultation with the geriatrics and gerontology teams. However, further studies are required, preferably longitudinal ones, to obtain a better critical assessment of the cutoff values of the tools used herein (VES-13 and WHOQOL-bref) to detect poorQoL.
